# Metagenomics reveals potential interactions between Patescibacteriota and their phages in groundwater ecosystems

**DOI:** 10.1128/msystems.01204-25

**Published:** 2025-12-23

**Authors:** Bingxin Hu, Liyun An, Mengdi Wu, Jinbo Xu, Yong Nie, Xiao-Lei Wu

**Affiliations:** 1 School of Mechanics and Engineering Science, Peking University 12465, Beijing, China; 2 School of Biological Science and Technology, University of Jinan 12413, Jinan, China; 3 School of Earth and Space Sciences, Peking University 12465, Beijing, China; 4 Institute of Ecology, Peking University 12465, Beijing, China; Institute of Biochemistry and Biophysics of the Polish Academy of Sciences, Warsaw, Poland

**Keywords:** phages, Patescibacteriota, phage-Patescibacteriota interactions, auxiliary metabolic genes

## Abstract

Patescibacteriota is a vast lineage composed of bacteria with ultra-small size, streamlined genomes, notable defects in core metabolic potential, and symbiotic lifestyle, which are widely detected in groundwater ecosystems. Increasing attention has focused on the physiological and ecological significance of Patescibacteriota, while the potential interactions between Patescibacteriota and their phages still need more exploration. Here, we collected 82 groundwater metagenomic data sets and further derived 1,162 phages with the potential to infect 2,439 groundwater Patescibacteriota metagenome-assembled genomes (MAGs). Notably, the groundwater Patescibacteriota MAGs were predominantly infected by temperate phages, and viral operational taxonomic unit/host Patescibacteriota operational taxonomic unit (OTU) abundance ratios were significantly negatively correlated with the relative abundance of host Patescibacteriota OTUs. Intriguingly, the groundwater Patescibacteriota phages encoded various auxiliary metabolic genes (AMGs) that might promote symbiotic lifestyle and metabolic potential of host Patescibacteriota MAGs. These included AMGs associated with concanavalin A-like lectin/glucanases superfamily and O-Antigen nucleotide sugar biosynthesis, which could enhance surface adhesion of host Patescibacteriota MAGs. Moreover, AMGs related to the ABC transport system and the P-type transporter could strengthen metabolic exchange and uptake of essential nutrients from the surroundings. Additionally, AMGs involved in various metabolic pathways might alleviate metabolic deficiencies in host Patescibacteriota MAGs.

**IMPORTANCE:**

Here, we sought phages that were capable of infecting Patescibacteriota metagenome-assembled genomes (MAGs), and further explored the diversity and novelty of Patescibacteriota phages, as well as the mechanisms underlying phage-Patescibacteriota interactions in groundwater ecosystems. The abundance profiles of phage-Patescibacteriota interactions suggested that lysogenic infection may represent a mutually adapted strategy between Patescibacteriota and their phages in groundwater ecosystems. Furthermore, the groundwater Patescibacteriota phages possessed diverse auxiliary metabolic genes which might facilitate the symbiotic associations and metabolic exchange between host Patescibacteriota MAGs and other free-living microbes and expand the metabolic capabilities of host Patescibacteriota MAGs. This study elucidated the mechanisms of phage-Patescibacteriota interactions and the potential roles of phages in modulating the physiology and ecology of Patescibacteriota within groundwater ecosystems.

## INTRODUCTION

Aquifers are essential components of the Earth’s deep terrestrial subsurface, also serving as significant natural reservoirs. They supply approximately 30% of the world’s freshwater (1), playing vital roles in drinking water provision, agricultural irrigation, and various industrial processes (2). The important roles of groundwater in human society and the Earth’s environment call for a deeper understanding of groundwater ecology and geochemistry (3). Despite having nutrient-limited oligotrophic conditions, groundwater ecosystems can still provide niches for various microbes (including bacteria and archaea) (4, 5). Groundwater microbial communities make great contributions to subsurface biogeochemical cycling of key elements (such as carbon, nitrogen, sulfur, phosphorus, and numerous metals) and energy conversion (6).

Notably, groundwater ecosystems with nutrient-limited and anoxic environmental conditions provide ideal habitats for a keystone taxon with unique evolutionary status and unclear ecological functions—Patescibacteriota (7–11). This monophyletic bacterial group is conventionally named by two widely used terms: Patescibacteria and candidate phyla radiation (CPR) (12). A recent study also proposed the phylum name “Minisyncoccota” for this bacterial group, following rules of the International Code of Nomenclature of Prokaryotes (ICNP) (13). However, in accordance with the phylum nomenclature adopted by Genome Taxonomy Database (GTDB) based on SeqCode recommendation (14), the taxonomic designation “Patescibacteriota” was employed to refer to this bacterial group in this study. Patescibacteriota comprise over 50% of all bacterial diversity (15). The discovery of Patescibacteriota has reshaped our understanding of the tree of life and underscored the critical roles of uncultured bacteria in the environment (16). Despite their high diversity, Patescibacteriota exhibit conserved traits, including ultra-small cell size, streamlined genomes, and minimal biosynthetic capabilities (17–19). Thus, Patescibacteriota adopt lifestyles relying on other cells, either by episymbiotically attaching to larger host bacteria to get nutrients or by deriving essential compounds from their surroundings (19).

Viruses are the most abundant, widespread, and genetically diverse biological entities in the biosphere, with a conservative estimate above 10^31^ (20). Recent studies have indicated that viruses widely inhabit aquatic (21–23), terrestrial (24), human-associated (25, 26), and engineered environments (27), even in extreme environments such as desert (28), glacier (29), permafrost (30), and acid mine drainage (31). Viruses profoundly influence the composition and evolution of microbial communities, playing crucial roles in various ecosystems (32, 33). Virulent viruses can regulate the composition of microbial communities by targeting and lysing the dominant populations, and contribute the host-derived organic matter to the surroundings through cell lysis, thereby affecting the global biogeochemical cycle (34). Moreover, viruses can regulate the metabolism and physiology of their host microbes via auxiliary metabolic genes (AMGs) (35). Virulent viruses exhibit AMGs associated with host metabolism to boost progeny reproduction, while temperate viruses tend to encode AMGs related to host physiological regulation to benefit virus-host coexistence (36). The ecological roles of virus-host interactions in different ecosystems require thorough contemplation.

Recently, some studies have focused on phages that can infect Patescibacteriota (37, 38). Due to the increasing relevance to human oral health and disease, Saccharibacteria, Gracilibacteria, and Absconditabacteria (SGA), lineages within Patescibacteriota, have attracted much attention. Liu et al. explored the diversity of CRISPR-Cas systems in SGA and sought phages with the potential to infect them by matching their CRISPR spacer inventories to phage databases (IMG/VR v3 and GVD Human Gut Virome). By examining the genetic code of predicted SGA phages, they found that some of them might infect both standard and alternatively coded host bacteria (37). Moreover, Wu et al. established the first metagenomic Groundwater Virome Catalogue and explored the diversity, lifestyle, and functional potential of phages connected to the keystone ultra-small symbionts (Patescibacteriota and DPANN archaea). They found that partial Patescibacteriota phages harbored genes associated with cell-surface modification that potentially assist symbiont cells adhere to free-living microbes (38). However, the mechanisms underlying phage-Patescibacteriota interactions, including the relationships between their relative abundance across different ecosystems and the roles of phages in supporting the symbiotic lifestyle and metabolic potential of Patescibacteriota through AMGs, remain underexplored. Here, we analyzed 82 groundwater metagenomic data sets that have been reported to contain abundant Patescibacteriota, and further explored the diversity and novelty of Patescibacteriota phages, as well as the mechanisms underlying phage-Patescibacteriota interactions in groundwater ecosystems.

## RESULTS

### Viruses are widely distributed in groundwater ecosystems

To uncover viruses in groundwater ecosystems, a total of 82 metagenomic data sets derived from sequential filtration of groundwater in Northern California and Colorado (7, 8, 11) were recruited and analyzed (Data S1). Raw sequencing reads were trimmed and assembled into contigs, then VirSoter2 was used to successfully recover 39,741 viral contigs that were ≥5 kb in size. Subsequently, all the viral contigs were clustered into 22,160 viral operational taxonomic units (vOTUs), representing approximately species-level taxonomy (Data S2). The genome size of vOTUs varied from 5 kb to 624 kb. A total of 2,543 vOTUs were estimated by CheckV to have genome completeness exceeding medium quality (Fig. S1). According to the taxonomic results based on the latest International Committee on Taxonomy of Viruses (ICTV) classification, 87.3% of the vOTUs could be assigned taxonomy (Fig. S2). At the class level, most (98.8%) of them were assigned to the class Caudoviricetes. Viruses in this class exhibit extremely high diversity and are widely distributed across various environments (20), with host ranges encompassing both bacteria and archaea. Additionally, the vast majority (97.1% and 97.5%, respectively) of vOTUs lacked taxonomic annotations at order or family level. Only 2.5% of vOTUs had taxonomic annotations at the family level, including Herelleviridae (0.83%), Mimiviridae (0.41%), Straboviridae (0.23%), Demerecviridae (0.13%), and Kyanoviridae (0.13%).

### Diversity and novelty of groundwater Patescibacteriota phages

**Fig 1:**
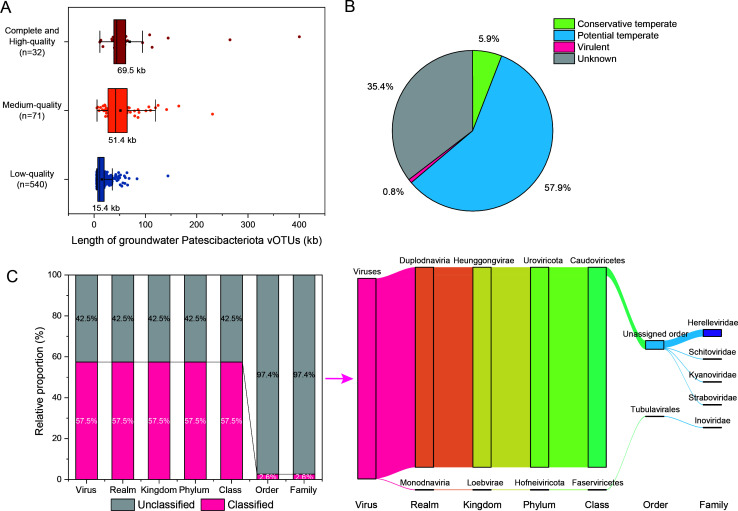
Overview of Patescibacteriota vOTUs identified in groundwater ecosystems. (**A**) Completeness and length of groundwater Patescibacteriota vOTUs based on CheckV assessment. For each box plot, the central line and the black square (with labeled value) indicate the median and the mean, respectively. The upper and lower bounds of boxes represent the interquartile range, spanning from the 25th to the 75th percentiles. The whiskers extend to 1.5× the interquartile range. (**B**) Lifestyle proportion of groundwater Patescibacteriota vOTUs. (**C**) Taxonomic affiliation of groundwater Patescibacteriota vOTUs based on the latest ICTV classification. Bar plot shows the proportion of classified (pink) and unclassified (gray) vOTUs. Sankey plot shows taxonomic affiliation of classified Patescibacteriota vOTUs.

To explore the potential impacts of phages on Patescibacteriota in groundwater ecosystems, the phage-Patescibacteriota interactions were investigated. Previous studies had proposed that most Patescibacteriota lack CRISPR-Cas systems (39), which play important roles in phage defense, while they might have alternative strategies to resist phage infection (10). Similarly, only 269 (9.64%) of the collected 2,790 groundwater Patescibacteriota metagenome-assembled genomes (MAGs) were predicted to possess CRISPR. Given this, three strategies (CRISPR spacers match, transfer RNA [tRNA] match, and nucleotide sequence homology search) were employed for phage-Patescibacteriota interactions (see Materials and Methods for more details; Fig. S3), ultimately generating 14,120 pairs of phage-Patescibacteriota interactions (Fig. S4A; Data S3) and uncovering 1,162 phages capable of infecting 2,438 host Patescibacteriota MAGs (Data S4 and S5). The groundwater Patescibacteriota phages were subsequently clustered into 644 Patescibacteriota vOTUs (Patescibacteriota vOTUs). The genome completeness of Patescibacteria vOTUs varied from fragmented sequences to complete genomes, with 16% of them exceeding medium quality (Fig. 1A). The genome size of Patescibacteria vOTUs ranged from 5 kb to 400 kb, with the majority ranging from 5 kb to 30 kb (Fig. 1A). Complete and high-quality Patescibacteria vOTUs had the largest mean size (69.5 kb), followed by medium quality (51.4 kb) and low quality (15.4 kb). As for the taxonomic classification, 57.5% of the Patescibacteriota vOTUs could be assigned taxonomic information (Fig. 1C). The vast majority (99.9%) of the Patescibacteriota vOTUs with taxonomic information were assigned to the class Caudoviricetes, with 4.3% of them having family-level taxonomic annotations, including Herelleviridae (3.5%), Schitoviridae (0.3%), Straboviridae (0.3%), and Kyanoviridae (0.3%). In terms of lifestyle, it was quite interesting that 63.8% of the Patescibacteriota vOTUs were considered as temperate phages (5.9% and 57.9% for conservative temperate and potential temperate, respectively), while only 0.8% of the Patescibacteriota vOTUs were inferred as virulent phages (see Materials and Methods for more details; Fig. 1B). Such a large ratio of temperate-to-virulent suggested that groundwater host Patescibacteriota MAGs were more prone to be infected by temperate phages, implying ingenious interactions between phages and host Patescibacteriota MAGs in groundwater ecosystems. Among the 411 temperate vOTUs identified in this paper, 92.7% were validated through detection within host Patescibacteriota MAGs using nucleotide sequence homology search (Fig. S5). Moreover, 55% of the conservative temperate Patescibacteriota vOTUs and 84% of the potential temperate Patescibacteriota vOTUs were predicted to be active (Fig. S6).

**Fig 2:**
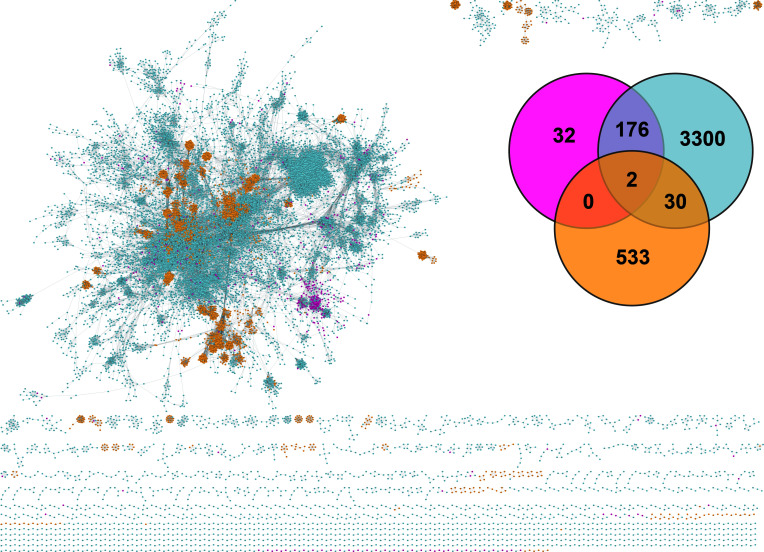
Relationship between vOTUs mined in this study and NCBI reference viral genomes based on gene-sharing network analysis. The nodes represent different viruses, while the shared edges indicate similarity based on shared protein clusters. Pink nodes represent groundwater Patescibacteriota vOTUs, blue nodes represent other vOTUs discovered in this study, while the orange nodes represent NCBI reference viral genomes. A Venn diagram shows the number of shared VCs among groundwater Patescibacteriota vOTUs, groundwater other vOTUs, and NCBI reference viral genomes.

To investigate the genomic similarity of groundwater Patescibacteriota vOTUs, other vOTUs mined in groundwater ecosystems, and publicly available sequences, a gene-sharing network analysis was performed using vConTACT2 to produce genus-level viral clusters (VCs) (Fig. 2). In general, 391 (60.71%) Patescibacteriota vOTUs, 11,804 (54.60%) groundwater other vOTUs, and 3,192 (91.15%) viral genomes from RefSeq v201 were grouped into 4,073 VCs. Among them, 32 VCs were exclusively comprised groundwater Patescibacteriota vOTUs, 3,300 VCs were solely formed by groundwater other vOTUs, and 533 VCs were purely composed of reference viral genomes. When assessing the genomic similarity between Patescibacteriota vOTUs and other vOTUs mined in groundwater ecosystems, it was found that only 289 (44.88%) Patescibacteriota vOTUs formed 178 VCs with other vOTUs, indicating a high degree of genomic novelty among Patescibacteriota vOTUs. Surprisingly, only two (0.31%) Patescibacteriota vOTUs could form two VCs with viral genomes from RefSeq v201, reflecting the huge and unexplored diversity of groundwater Patescibacteriota vOTUs.

### Close interactions between host Patescibacteriota MAGs and their phages in groundwater ecosystems

**Fig 3:**
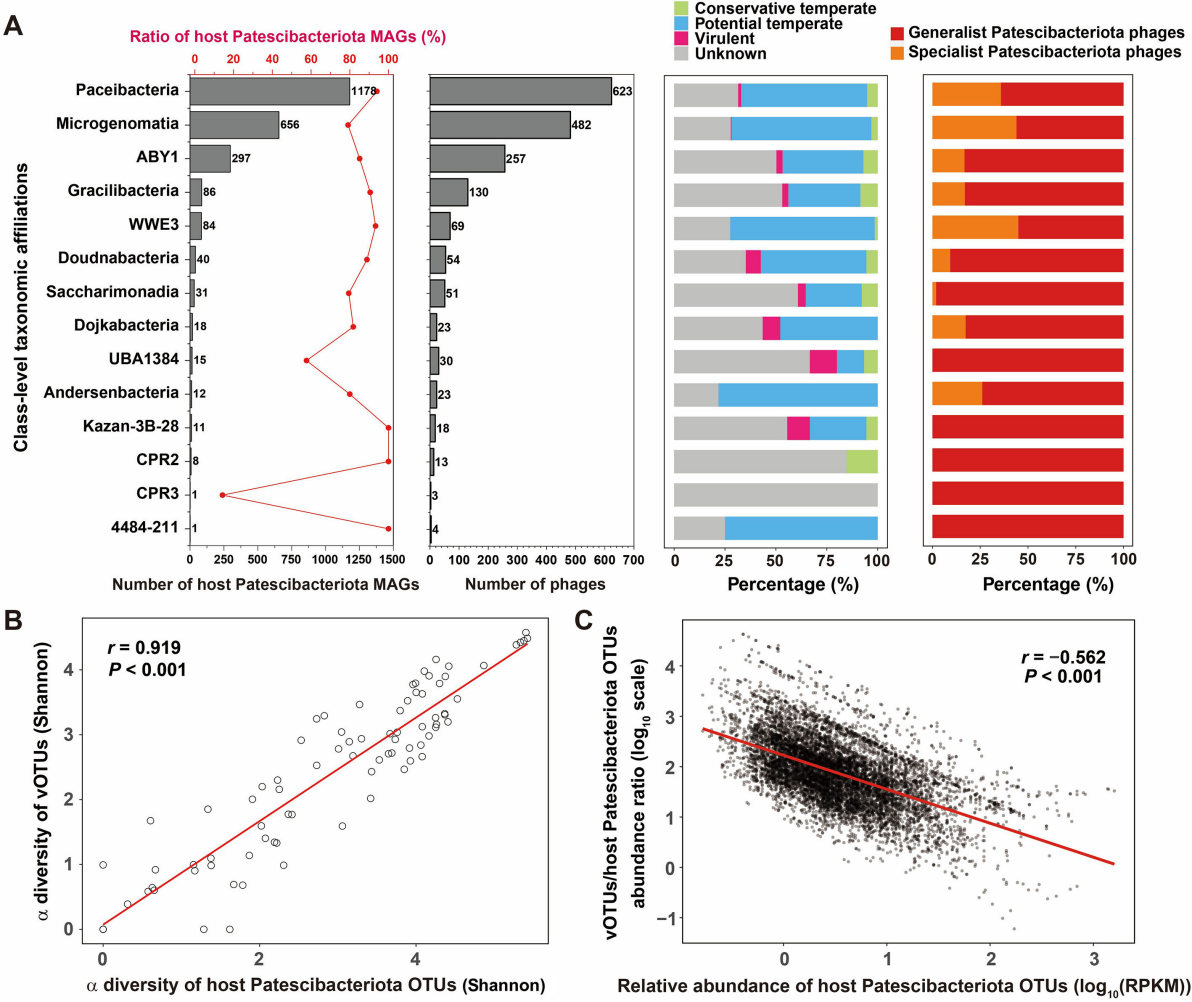
Interactions between host Patescibacteriota MAGs and their phages in groundwater ecosystems. (**A**) Number and ratio of host Patescibacteriota MAGs, number of phages, lifestyle ratio of phages, and the ratio of generalist and specialist phages for each class-level taxonomic affiliations. (**B**) Comparisons of α-diversity (Shannon index) between host Patescibacteriota OTUs and their vOTUs. (**C**) Correlation between the relative abundance of host Patescibacteriota OTUs and vOTUs/host Patescibacteriota OTUs abundance ratios.

To better understand the complex interactions between phages and host Patescibacteriota MAGs in groundwater ecosystems, both the capability of phages to infect host Patescibacteriota MAGs and the susceptibility of host Patescibacteriota MAGs to phage infection were examined. For the 1,162 Patescibacteriota phages, 547 (47.1%) phages were exclusively connected to a unique host Patescibacteriota MAG (termed specialist Patescibacteriota phages), while 615 (52.9%) phages were connected to more than one host Patescibacteriota MAG (termed generalist Patescibacteriota phages) (Fig. S4B). For 2,438 host Patescibacteriota MAGs, only 310 (12.7%) host Patescibacteriota MAGs were connected to a certain Patescibacteriota phage (termed specialist host Patescibacteriota MAGs), while 2,128 (87.3%) host Patescibacteriota MAGs were connected to more than one Patescibacteriota phage (termed generalist host Patescibacteriota MAGs) (Fig. S4C). The host Patescibacteriota MAGs were distributed across 14 classes, which were highly represented by the class Paceibacteria (*n* = 1,178), Microgenomatia (*n* = 656), ABY1 (*n* = 297), Gracilibacteria (*n* = 86), and WWE3 (*n* = 84). With the exception of UBA1384 and CPR3, more than 75% of Patescibacteriota MAGs from other classes were predicted to be the host of phages (Fig. 3A). Phages connected to each class-level taxonomic affiliations of host Patescibacteriota MAGs were dominated by temperate phages and generalist Patescibacteriota phages (Fig. 3A).

To further investigate the interactions between host Patescibacteriota MAGs and their phages in groundwater ecosystems, we deduplicated these interactions to establish linkages between host Patescibacteriota OTUs (operational taxonomic units) and their vOTUs. Subsequently, a correlation analysis was performed on the profiles between host Patescibacteriota OTUs and their vOTUs. There was a significantly positive correlation between the α-diversity (Shannon diversity) of host Patescibacteriota OTUs and their vOTUs, indicating that their community structures were closely related in groundwater ecosystems (*r* = 0.919, *P* < 0.001; Fig. 3B). Additionally, similar to VHR (viral-to-host ratios), vOTUs/host Patescibacteriota OTUs abundance ratios were significantly negatively correlated with the relative abundance of host Patescibacteriota OTUs (*r* = −0.562, *P* < 0.001; Fig. 3C). With the increase of the relative abundance of host Patescibacteriota OTUs, vOTUs/host Patescibacteriota OTUs abundance ratios decreased, resulting in the phenomenon of “more host microbes, fewer phages.” This result suggested that more Patescibacteriota phages will choose to exploit their hosts by lysogenic infection rather than lytic infection at high microbial abundance (40). Temperate phages can protect their hosts from being infected by other phages via superinfection exclusion (41) or alter the physiology of their host microbes via AMGs (35), thereby enhancing the fitness and competitiveness of their host microbes. Therefore, we speculated that when the abundance of host Patescibacteriota OTUs in groundwater ecosystems is high, their vOTUs tend to enhance lysogenic infection to help them sustain the dominance in the microbial communities and maintain stable phage-Patescibacteriota coexistence. By analyzing six trimmed metatranscriptomic data sets from the Colorado site (Data S1), we found that vOTUs/host Patescibacteriota OTUs activity ratios were significantly negatively correlated with the relative activity of host Patescibacteriota OTUs (*r* = −0.391, *P* < 0.001; Fig. S7).

### Phages potentially promote symbiotic associations and metabolic exchange between host Patescibacteriota MAGs and other free-living microbes

To further investigate how the groundwater Patescibacteriota phages might affect host Patescibacteriota MAGs, 355 phage-encoded putative AMGs were predicted using DRAM-v (Data S6). Various indications suggest that Patescibacteriota adopt a lifestyle that relies on other cells, either by deriving essential compounds from surroundings or by episymbiotically attaching to larger host microbes to get nutrients (19).

**Fig 4:**
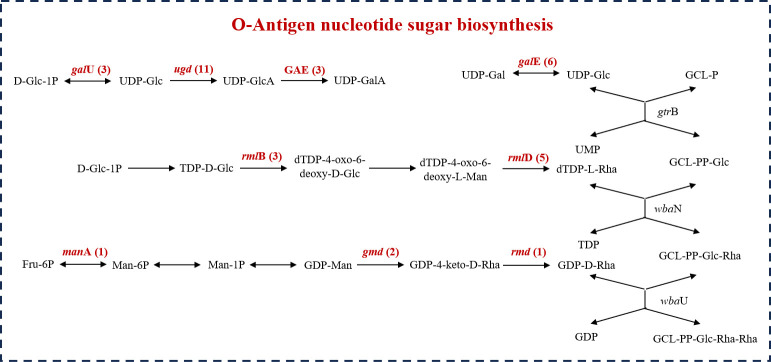
Putative AMGs identified in groundwater Patescibacteriota phages involved in O-Antigen nucleotide sugar biosynthesis. The phage-encoded AMGs are highlighted in red within these pathways, with Arabic numerals in parentheses indicating their respective quantities. GCL, glycosyl-carrier lipid.

Considering the requirement for attachment to other free-living microbes, it is unsurprising that Patescibacteriota genomes typically encode numerous proteins associated with diverse cell-surface modifications, including pili, glycosyltransferase, and concanavalins (lectins) (19). Interestingly, the groundwater Patescibacteriota phages harbored five AMGs annotated as concanavalin A-like lectin/glucanases superfamily (Data S6). These proteins have been speculated to be involved in surface attachment of Patescibacteriota to other free-living microbes (38). What’s more, the groundwater Patescibacteriota phages possessed 46 AMGs involved in O-Antigen nucleotide sugar biosynthesis, including genes encoding UDP-glucose 4-epimerase (*gal*E), dTDP-glucose 4,6-dehydratase (*rml*B), dTDP-4-dehydrorhamnose reductase (*rml*D), mannose-6-phosphate isomerase (*man*A), GDP-mannose 4,6-dehydratase (*gmd*), and GDP-4-dehydro-6-deoxy-D-mannose reductase (*rmd*) (Fig. 4; Data S6). The same as previous research (42), these AMGs encoded by phages may enrich rhamnose in the outer membrane lipopolysaccharide of host Patescibacteriota MAGs by converting dTDP-4-oxo-6-deoxy-L-mannose to dTDP-L-rhamnose, and GDP-D-mannose to GDP-D-rhamnose, potentially contributing to surface adhesion and cell-cell aggregation of host Patescibacteriota MAGs, thereby promoting symbiotic associations between host Patescibacteriota MAGs and other free-living microbes.

Moreover, Patescibacteriota genomes encode numerous transporters to fulfill the need for deriving essential compounds from the surroundings (19). It was ingenious that the groundwater Patescibacteriota phages encoded various AMGs associated with transport systems, mainly including the ABC transport system and the P-type transporter (Table S1). ABC transport system utilizes the energy derived from ATP hydrolysis to actively transport nutrients (such as sugars, amino acids, and inorganic ions) across concentration gradients into bacterial cells (43). These AMGs encoding various transporters might also enhance the metabolic exchange between host Patescibacteriota MAGs and other free-living microbes.

Besides, the groundwater Patescibacteriota phages also encoded P-type transporters, which can utilize the energy generated by ATP hydrolysis to transport specific ions (such as Ca^2+^, Mg^2+^, Cu^2+^, Cu^+^, etc.) across membranes (44), making them crucial for regulating ion concentration and osmotic pressure across the cell membrane. These ions also serve as essential cofactors for certain enzymes involved in bacterial growth and metabolism (45). In oligotrophic groundwater ecosystems, through AMGs associated with transporters, phages might not only enhance the uptake of essential nutrients from surroundings, but also maintain ionic homeostasis across the cell membrane, thereby enhancing the environmental adaptability and survival ability of host Patescibacteriota MAGs.

### Phages potentially expand metabolic capabilities of host Patescibacteriota MAGs

Moreover, the groundwater Patescibacteriota phages tended to encode AMGs associated with metabolism, mainly involved in carbohydrate metabolism (*n* = 113), amino acid metabolism (*n* = 85), metabolism of cofactors and vitamins (*n* = 68), glycan biosynthesis and metabolism (*n* = 56), energy metabolism (*n* = 55), and nucleotide metabolism (*n* = 43) based on the KEGG database (Fig. S8).

**Fig 5:**
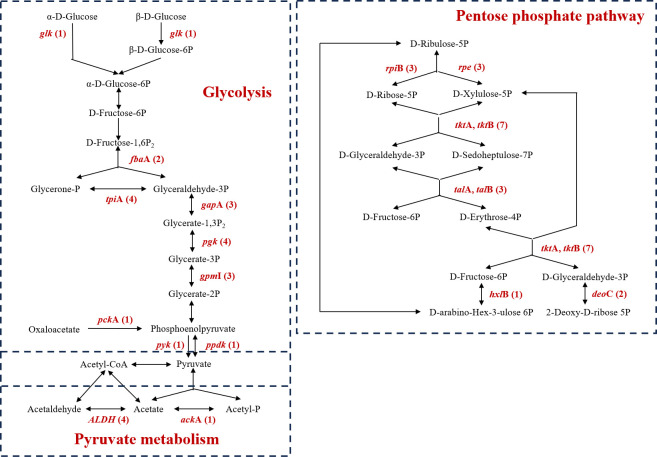
Putative AMGs identified in groundwater Patescibacteriota phages involved in glycolysis, pentose phosphate pathway, and pyruvate metabolism. The phage-encoded AMGs are highlighted in red within these pathways, with Arabic numerals in parentheses indicating their respective quantities.

For carbohydrate metabolism, five AMGs were annotated as concanavalin A-like lectin/glucanases superfamily, which was associated with phage-encoded glycoside hydrolase, potentially involved in pectin cleavage (29). The carbon sources for Patescibacteriota typically consist of complex carbon compounds derived from degraded plant or microbial biomass (19). Therefore, the ability to degrade these complex compounds into simpler monomeric forms is essential for them. By cleaving polymers into monomers, these glycoside hydrolases may facilitate carbon utilization of host Patescibacteriota MAGs in oligotrophic groundwater ecosystems. What’s more, the groundwater Patescibacteriota phages encoded various AMGs involved in glycolysis, pentose phosphate pathway, and pyruvate metabolism (Fig. 5). Most of the Patescibacteriota lack complete glycolysis and pentose phosphate pathway, usually compensating for the impaired central carbon metabolism through metabolic shunt (19). A previous study suggested that viruses could compensate for the incomplete central carbon metabolism (mainly pentose phosphate pathway) of DPANN archaea through AMGs (36). This inspired our hypothesis that phages might significantly enhance the central carbon metabolism capacity of host Patescibacteriota MAGs via AMGs, reducing their dependence on metabolic shunt. Certain Patescibacteriota are abundant in acetate-amended groundwater, implying their ability to utilize acetate (7, 8). Phage-carried AMGs encoding acetate kinase and aldehyde dehydrogenase might reversely promote acetate utilization, potentially explaining the proliferation of host Patescibacteriota MAGs following acetate stimulation.

For the metabolism of cofactors and vitamins, the groundwater Patescibacteriota phages encoded various AMGs involved in nicotinate and nicotinamide metabolism, folate biosynthesis, and one-carbon pool by folate (Fig. S9). Nicotinate and nicotinamide are precursors of NAD, which act as electron carriers in glycolysis, the tricarboxylic acid cycle, and oxidative phosphorylation, participating in energy generation and conversion (46). Folate is an important cofactor that mediates the transfer of one carbon unit (47). It participates in the *de novo* synthesis of purines and pyrimidines, as well as the synthesis of various amino acids (glycine, serine, methionine, etc.), making it crucial for the growth and proliferation of bacterial cells. There were also two AMGs identified as cysteine desulfurase, which could decompose L-cysteine to L-alanine and sulfane sulfur (48), thus potentially involved in the biosynthesis of a variety of cofactors (including Fe-S clusters in proteins, thionucleosides in tRNA, thiamine, biotin, lipoic acid, molybdopterin, and NAD) (Data S6). Many enzymes encoded in Patescibacteriota require cofactors, yet pathways for their biosynthesis were rarely identified (19). In this context, phage-encoded AMGs might partially alleviate the challenges host Patescibacteriota MAGs face in cofactors and vitamins metabolism.

For energy metabolism, there were five AMGs involved in oxidative phosphorylation, encoding F-type H^+^/Na^+^-transporting ATPase subunit alpha, beta, and gamma (Data S6). F-type H^+^/Na^+^-transporting ATPase utilizes transmembrane proton gradient to drive the production of ATP, which serves as the primary energy source for various biological processes in bacterial cells (49). Additionally, the groundwater Patescibacteriota phages encoded numerous AMGs involved in amino acid metabolism and nucleotide metabolism (Fig. S10; Data S6). Patescibacteriota appear to have defects in the biosynthesis of amino acids and nucleotides, often needing to salvage them from external sources (19). However, phage-encoded AMGs might alleviate these deficiencies and reduce their dependence on the surroundings.

## DISCUSSION

A previous study has proposed the widespread absence of CRISPR-Cas systems in Patescibacteriota and has suggested that CRISPR-Cas systems are probably selected against by Patescibacteriota because the costs of maintaining the systems outweigh the benefit they provide (39). However, cryogenic transmission electron microscopy (cryo-TEM) characterization in previous studies has detected virus particles directly associated with the surfaces of Patescibacteriota (50), providing evidence for phage-Patescibacteriota interactions. Based on previous findings, three strategies were used to mine phages capable of infecting Patescibacteriota MAGs from 82 groundwater metagenomic data sets. The taxonomic assignment and gene-sharing network analysis indicated rich diversity and great novelty of the groundwater Patescibacteriota vOTUs. In groundwater ecosystems, host Patescibacteriota OTUs and their vOTUs formed intricate interactions. We also identified diverse AMGs in Patescibacteriota phages, which might facilitate the symbiotic associations and metabolic exchange between host Patescibacteriota MAGs and other free-living microbes and expand the metabolic capabilities of host Patescibacteriota MAGs.

Viral lifestyle strategies are central to the ecology of virus-host interactions, mainly through the lytic and lysogenic replication cycles, referring to “Kill the Winner” (KtW) hypothesis and “Piggyback-the-Winner” (PtW) hypothesis, respectively (40). Lytic infection leads to the reproduction of viral progeny, the lysis of host cells, and the release of the cellular dissolved organic matter into the surrounding environment (34). The corresponding KtW hypothesis suggests that higher host microbial abundance is associated with stronger lytic infection, leading to higher VHR (35). In this case, viruses increase the availability of resources by persistently killing the dominant microbes, promoting the growth of relatively disadvantaged species, thereby enhancing the diversity of the microbial community (40). Lysogenic infection is chosen by temperate viruses to integrate into, and replicate with, the genome of host microbes by forming a mutualistic relationship (51). The corresponding PtW hypothesis suggests that higher host microbial abundance enhances lysogenic infection and reduces VHR (41). In this context, viruses confer competitive advantages to the dominant microbes through superinfection exclusion or AMGs, inhibiting the growth of relatively disadvantaged species, thereby reducing the diversity of the microbial community (40). We found that host Patescibacteriota MAGs were predominantly infected by temperate phages in groundwater ecosystems, and the abundance profiles between host Patescibacteriota OTUs and vOTUs aligned with the pattern exhibited by the PtW hypothesis. However, we still lack sufficient evidence to prove that the interactions between host Patescibacteriota OTUs and vOTUs follow the PtW hypothesis, necessitating further experimental studies for validation. Given that groundwater ecosystems are typical oligotrophic habitats, and Patescibacteriota had high relative abundance in the collected groundwater samples, these findings are consistent with the previously reported view that lysogenic infection was generally considered to be more prevalent and important in harsh environments (35, 40). From the perspective of phages, the lack of CRISPR in Patescibacteriota provides opportunities and convenience for their lysogenic infection. From the perspective of Patescibacteriota, they face a challenge of relying on external resources for survival due to deficiencies in core metabolic capabilities. Therefore, they need to enhance lysogenic infection to maximize their competitive advantages, thus enabling them to maintain dominance in the microbial communities. Overall, lysogenic infection may represent a strategy that has been gradually selected during the coexistence of Patescibacteriota and their phages in groundwater ecosystems.

**Fig 6:**
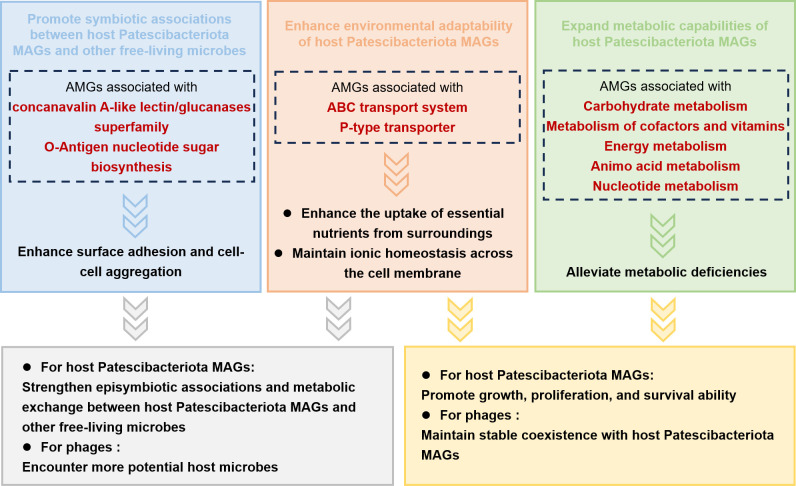
Schematics of the impact of AMGs on the interactions between host Patescibacteriota MAGs and their phages in groundwater ecosystems.

Groundwater ecosystems are typical oligotrophic habitats with nutrients in low concentration and limited diversity (52), driving Patescibacteriota residing in these habitats to streamline their metabolic capabilities and biosynthetic pathways. A previous study suggested that most of the genomes of Patescibacteriota lacked complete glycolysis and pentose phosphate pathway, compensating for the impaired central carbon metabolism through metabolic shunt (19). Moreover, due to the lack of complete biosynthetic pathways for cofactors, amino acids, and nucleotides, many Patescibacteriota need to acquire these nutrients from external sources (19). Intriguingly, we identified various AMGs in Patescibacteriota phages, which might play interesting roles in the interactions between host Patescibacteriota MAGs and their phages in groundwater ecosystems (Fig. 6). As symbionts that rely on other free-living microbes for essential nutrients, Patescibacteriota can attach to other larger microbes through various cell-surface modifications (19). The groundwater Patescibacteriota phages encoded numerous AMGs associated with concanavalin A-like lectin/glucanases superfamily and O-Antigen nucleotide sugar biosynthesis, which could enhance surface adhesion of host Patescibacteriota MAGs. These AMGs, along with AMGs associated with the ABC transport system and P-type transporter, might strengthen episymbiotic associations and metabolic exchange between host Patescibacteriota MAGs and other free-living microbes. This would, in return, increase the likelihood of Patescibacteriota phages encountering potential host microbes, thus enhancing their infectivity. The groundwater Patescibacteriota phages also harbored numerous AMGs, which not only alleviate metabolic deficiencies in host Patescibacteriota MAGs but also enhance their uptake of essential nutrients from the surroundings. With the assistance of these AMGs, the growth, proliferation, and survival ability of host Patescibacteriota MAGs would be promoted, thereby maintaining stable phage-Patescibacteriota coexistence. We calculated the relative activity and the relative abundance of AMGs carried by the groundwater Patescibacteriota phages in six metatranscriptomic data sets and their corresponding metagenomic data sets from the Colorado site. Several AMGs were transcriptionally active *in situ*, involved in carbohydrate metabolism, energy metabolism, nucleotide metabolism, and amino acid metabolism (Fig. S11). However, other AMGs, despite their high relative abundance in metagenomes, exhibited no detectable transcriptional activity in metatranscriptomes. This discordance may arise from technical constraints encompassing RNA capture biases and sequencing depth limitations. Further laboratory and *in situ* experiments are required to validate these findings.

In conclusion, our study revealed the diversity and novelty of phages with the potential to infect host Patescibacteriota MAGs in groundwater ecosystems. We elucidated the complex interactions between host Patescibacteriota MAGs and their phages and found that phages potentially promoted the symbiotic lifestyle and metabolic potential of host Patescibacteriota MAGs through AMGs. These results provide valuable insights into the ecological roles of phage-Patescibacteriota interactions in groundwater ecosystems.

## MATERIALS AND METHODS

### Data acquisition and processing

Based on three previous studies (7, 8, 11), a total of 82 groundwater metagenomic data sets were downloaded from NCBI Sequence Read Archive and NCBI BioSample databases (Data S1). Raw reads were trimmed using the Trimmomatic v0.3962 (53) (parameters: LEADING:2 TRAILING:2 SLIDINGWINDOW:4:20 MINLEN:50), followed by individual assembly using SPAdes v3.15.4 (54, 55) (parameters: -meta, -k 21,33,55,77,99,127) or MEGAHIT v1.2.9 (56) (default parameters). Six trimmed metatranscriptomic data sets from the Colorado site were also downloaded and subsequently removed rRNA reads using sortmerna v4.3.4 (57) (parameters: -fastx -blast 1 -num_alignments 1). Moreover, MAGs binned from the 82 groundwater metagenomic data sets were also downloaded. After taxonomic assignment using GTDB-Tk v1.5.0 (58) based on GTDB (http://gtdb.ecogenomic.org), 2,790 Patescibacteriota MAGs were retained for further analysis.

### Recovery of groundwater viral contigs

Viral contigs were recovered from assembled contigs using VirSorter v2.1 (59) (parameters: --exclude-lt2gene), and only viral contigs ≥ 5 kb were retained. Completeness of the initial viral contigs was estimated using the CheckV v0.8.1 (60) pipeline (end_to_end program). The final viral contigs were obtained by merging the output file of CheckV (“viruses.fna” and “proviruses.fna”), which had removed contamination of host microbes. Any viral contigs annotated as “no viral genes” were also manually discarded. Completeness and length of the final viral contigs were also estimated using the CheckV v0.8.1 pipeline (end_to_end program). The groundwater viral contigs were further clustered into vOTUs with parameters of 95% average nucleotide identity (ANI) and 85% alignment fraction based on scripts provided in CheckV v0.8.1 (Data S2; https://bitbucket.org/berkeleylab/checkv/src/master/).

### Identification of putative groundwater Patescibacteriota phages

Three strategies were used to find out phages with the potential to infect groundwater Patescibacteriota MAGs, including CRISPR spacers match, transfer RNA (tRNA) match, and nucleotide sequence homology search (21, 61, 62). (i) CRISPR spacer match: the CRISPR was searched from 2,790 Patescibacteriota MAGs using metaCRT (63) (modified from CRT1.2) with default parameters. Extracted CRISPR spacers were further matched against groundwater viral contigs using BLASTn (64) (parameters: E-value ≤ 1E−5, percentage identity of 95%, mismatches ≤1). (ii) tRNA match: the tRNA genes contained in groundwater viral contigs were identified by ARAGORN v1.2.38 (65) with the “−t” option, further aligned with the 2,790 Patescibacteriota MAGs using BLASTn (64) (90% coverage and 90% identity). (iii) Nucleotide sequence homology search: groundwater viral contigs were searched against 2,790 Patescibacteriota MAGs based on shared genomic regions using BLASTn (64) (thresholds: 75% minimum coverage of the viral contig length, 70% minimum nucleotide identity, 50 minimum bit score, and 0.001 maximum e-value). Previous studies suggested that this method yielded prophages (21). The groundwater Patescibacteriota phages were further clustered into Patescibacteriota vOTUs using the same method mentioned above. All the host Patescibacteriota MAGs were de-replicated at 95% ANI using dRep v3.2.2 (66) to generate estimated species-level host Patescibacteriota OTUs (parameters: -comp 50 -con 10 -nc 0.30 -pa 0.9 -sa 0.95).

### Taxonomic assignment and lifestyle prediction of groundwater Patescibacteriota phages

All the groundwater vOTUs (including those connected to host Patescibacteriota MAGs) were further annotated using geNomad v1.7.3 (67) based on ICTV classification to get the taxonomic assignment.

Referring to a previous study (38), the lifestyle of the groundwater Patescibacteriota vOTUs was predicted using a strategy that combined the results of geNomad (67), CheckV (60), BACPHLIP (68), and nucleotide sequence homology search (21). (i) For all the Patescibacteriota vOTUs, integrated prophages identified by both geNomad and CheckV were considered as conservative temperate phages. (ii) For complete and high-quality Patescibacteriota vOTUs, those with a probability above 90% in BACPHLIP predictions were considered as conservative temperate phages and virulent phages, respectively. (iii) Other Patescibacteriota vOTUs that were connected to host Patescibacteriota MAGs based on nucleotide sequence homology search were considered as potential temperate phages. (iv) The lifestyle of the remaining Patescibacteriota vOTUs was regarded as unknown.

The potential activity of temperate Patescibacteriota vOTUs was predicted using PropagAtE v1.1.0 with default parameters (69).

### Gene-sharing network analysis

The sequences of vOTUs recovered in this study, including the groundwater Patescibacteriota vOTUs, were pooled to call open reading frames using Prodigal v2.6.3 (70) (parameters: -p meta -f gff -q -m). The resulting protein sequences were further input into vConTACT2 v2.086 (71) to run against the NCBI Prokaryotic Viral RefSeq v201 database (parameters: --rel-mode Diamond --pcs-mode MCL --vcs-mode ClusterONE). Phages were clustered based on the similarity of the shared protein clusters. The resulting network was visualized in Cytoscape v3.9.1 (72) (http://cytoscape.org).

### Abundance and activity profiles

RPKM (reads per kilobase per million mapped reads) were used to represent the relative abundance of host Patescibacteriota OTUs and their vOTUs. The RPKM of vOTUs were calculated using CoverM v0.6.1 (https://github.com/wwood/CoverM) to generate coverage profiles across samples (parameters: coverm contig --min-read-percent-identity 0.95, --min-read-aligned-percent 0.75, --contig-end-exclusion 0, and -m rpkm). Similarly, the RPKM of host Patescibacteriota OTUs were also calculated using CoverM v0.6.1 to generate coverage profiles across samples (parameters: coverm genome --min-read-percent-identity 0.95, --min-read-aligned-percent 0.75, --contig-end-exclusion 0, and -m rpkm). Similar to VHR, vOTUs/host Patescibacteriota OTUs abundance ratios were calculated by dividing the relative abundance of vOTUs by the relative abundance of host Patescibacteriota OTUs. CoverM v0.6.1 was also used to calculate the relative activity of host Patescibacteriota OTUs and their vOTUs, with parameters consistent with those described above‌ (except that --min-covered-fraction was set to 0). vOTUs/host Patescibacteriota OTUs activity ratios were calculated by dividing the relative activity of vOTUs by the relative activity of host Patescibacteriota OTUs.

### Identification of AMGs

All the 1,162 groundwater Patescibacteriota phages were rerun by VirSorter v2.1 (--prep-for-dramv) to generate the output files that DRAM-v required (61, 73). Then the DRAM-v v1.3.5 (74) was used to recover putative AMGs. To be more conservative, only putative AMGs with an auxiliary score < 4 were retained. Putative AMGs without gene descriptions were further annotated using GhostKOALA to get functional annotations (https://www.kegg.jp/ghostkoala/). In addition, putative AMGs associated with ribosome, peptidase, and proteasome, or still without gene descriptions, were discarded. Subsequently, a manual curation process was undertaken by checking the presence of viral hallmark genes or virus genes upstream and downstream of the selected AMGs. The relative abundance and the relative activity of AMGs were calculated using CoverM with identical parameters as previously described.

## Data Availability

NCBI accession numbers for metagenome reads used in this study (BioProject: PRJNA640378, PRJNA288027, and PRJNA268031) are provided in Data S1. Metagenome-assembled genomes were retrieved from assembly datasets of PRJNA640378, PRJNA288027, and PRJNA273161.

## SUPPLEMENTAL MATERIAL

Supplemental figures and table msystems.01204-25-s0001.docx Fig. S1 to S11; Table S1.

Supplemental Data msystems.01204-25-s0002.xlsx Data S1 to S6.

ASM does not own the copyrights to Supplemental Material that may be linked to, or accessed through, an article. The authors have granted ASM a non-exclusive, world-wide license to publish the Supplemental Material files. Please contact the corresponding author directly for reuse.
