# Supplementary material for: Metagenomics reveals potential interactions between Patescibacteriota and their phages in groundwater ecosystems
Source: mSystems. 2025 Dec 23;11(2):e01204-25. doi: 10.1128/msystems.01204-25 (PMC12911398; doi:10.1128/msystems.01204-25)
Supplement: Supplemental figures and table — Fig. S1 to S11; Table S1. [file msystems.01204-25-s0001.docx]

Supplementary materials for

**Metagenomics reveals potential interactions between Patescibacteriota and their phages in groundwater ecosystems**

Bingxin Hu^1^, Liyun An^2^, Mengdi Wu^3^, Jinbo Xu^3^, Yong Nie^1*^, Xiao-Lei Wu^1,4*^

^1^ School of Mechanics and Engineering Science, Peking University, Beijing 100871, China

^2^ School of Biological Science and Technology, University of Jinan, Jinan 250022, China

^3^ School of Earth and Space Sciences, Peking University, Beijing 100871, China

^4^ Institute of Ecology, Peking University, Beijing 100871, China

*Corresponding author:

Research Scientist, School of Mechanics and Engineering Science, Peking University.

Tel: +86 10-62759047; Fax: +86 10-62759047; E-mail: nieyong@pku.edu.cn

*Corresponding author:

Professor, School of Mechanics and Engineering Science, Peking University.

Tel: +86 10-62759047; Fax: +86 10-62759047; E-mail: xiaolei_wu@pku.edu.cn

# Supplementary Figures


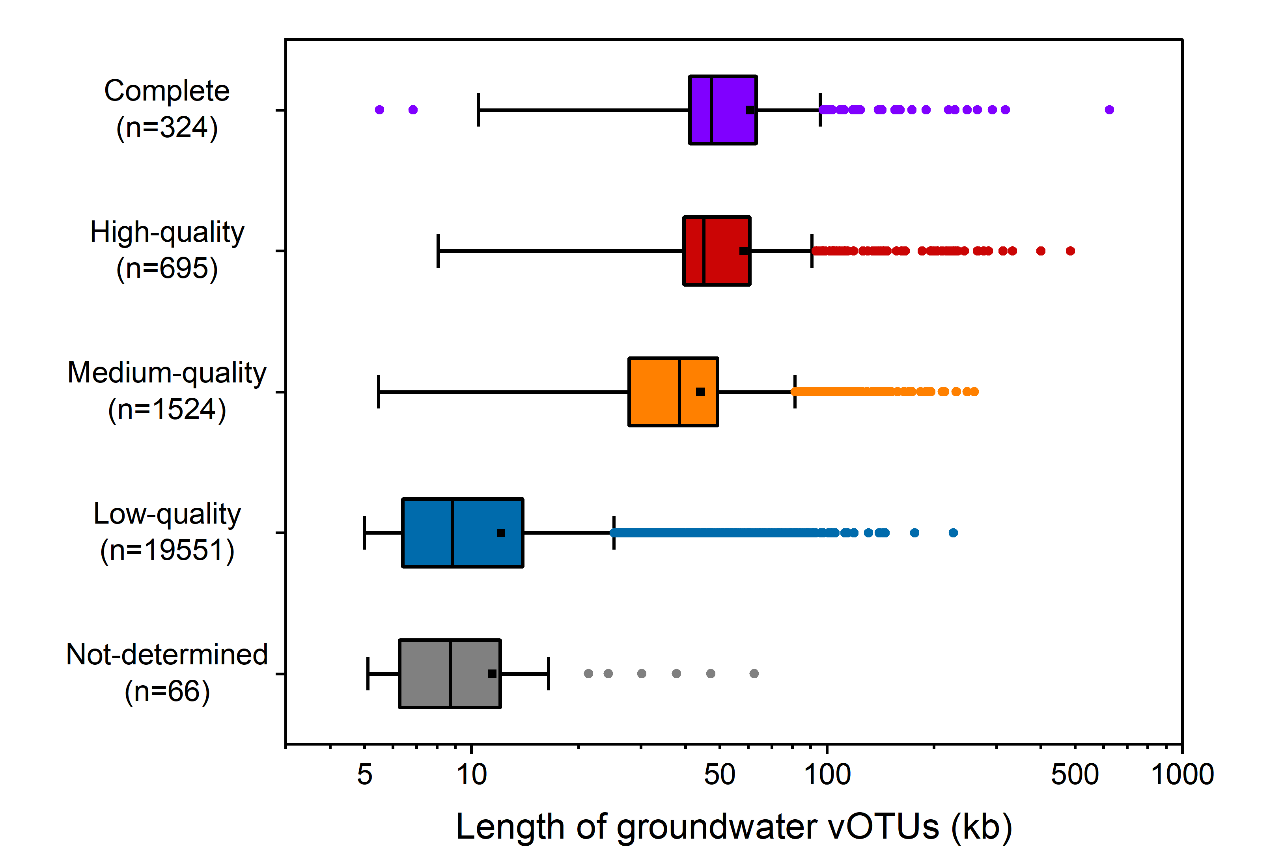


Figure S1. Completeness and length of groundwater vOTUs based on CheckV assessment. For each box plot, the central line and the black square indicate the median and the mean, respectively. The upper and lower bounds of boxes represent the interquartile range, spanning from the 25th to the 75th percentiles. The whiskers extend to 1.5 times the interquartile range.


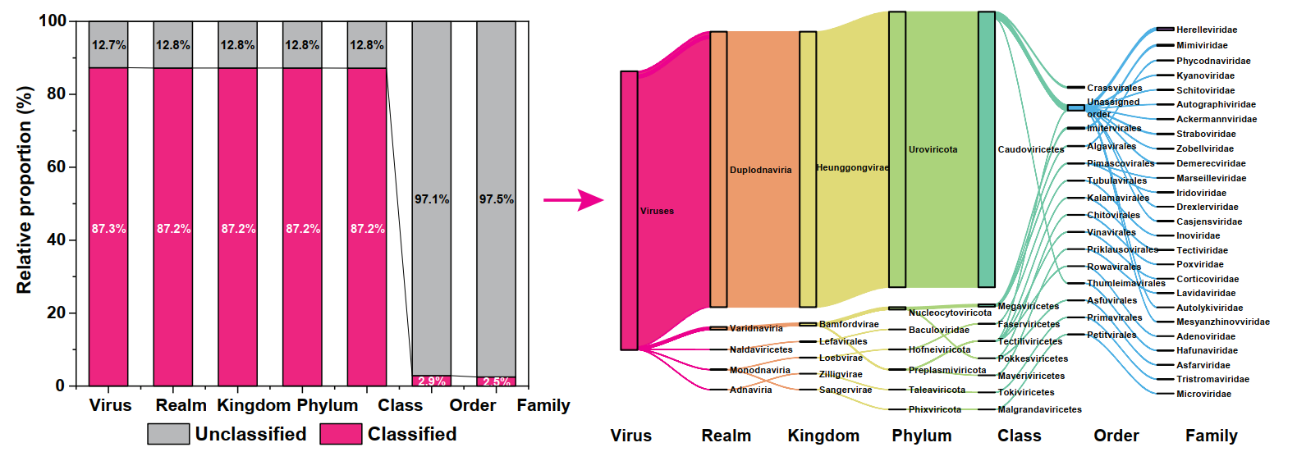


Figure S2. Taxonomic affiliation of vOTUs mined in this study based on the latest ICTV classification. Bar plot shows the proportion of classified (pink) and unclassified (grey) vOTUs. Sankey plot shows viral taxonomic affiliation of classified vOTUs.


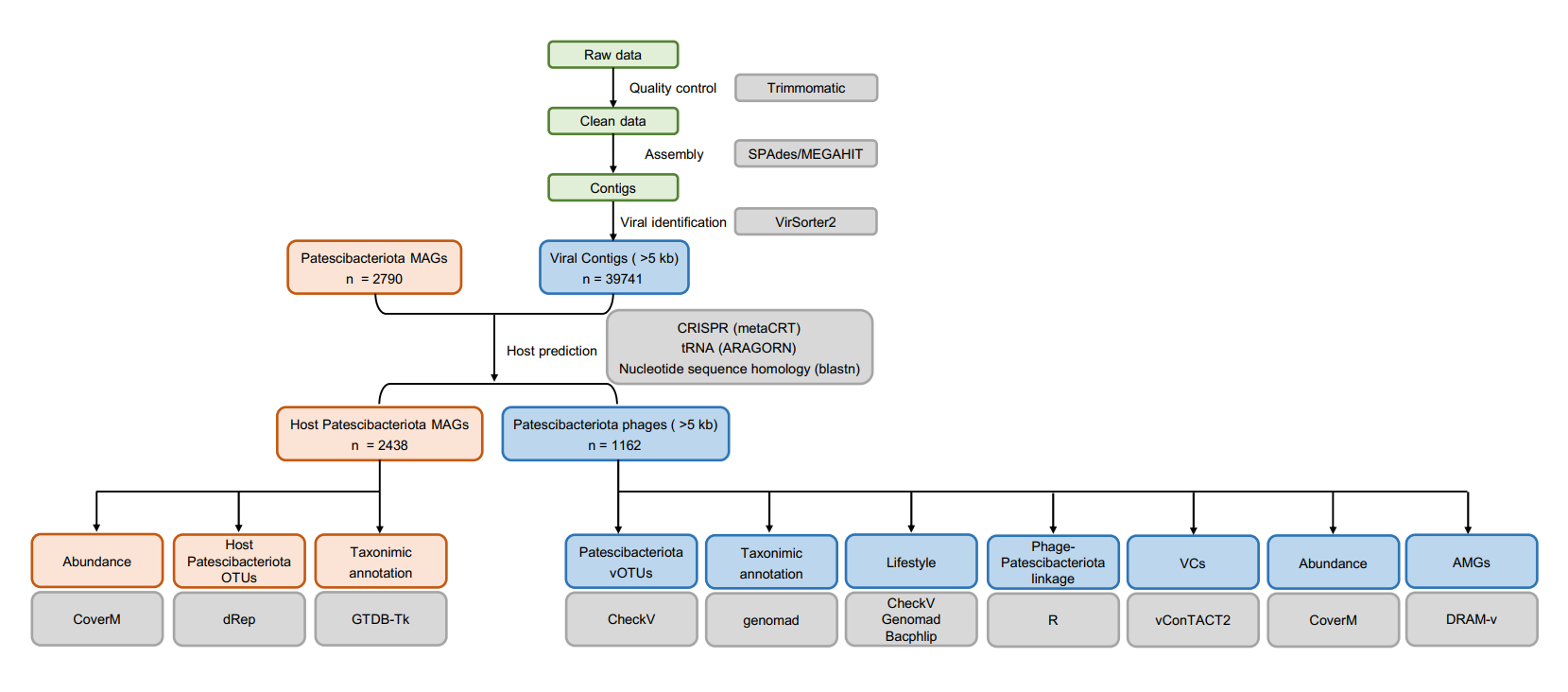


Figure S3. Bioinformatic analysis workflow for groundwater Patescibacteriota phages. Green: data collection and initial processing; orange: analysis of Patescibacteriota MAGs; blue: analysis of Patescibacteriota phages; grey: software used.


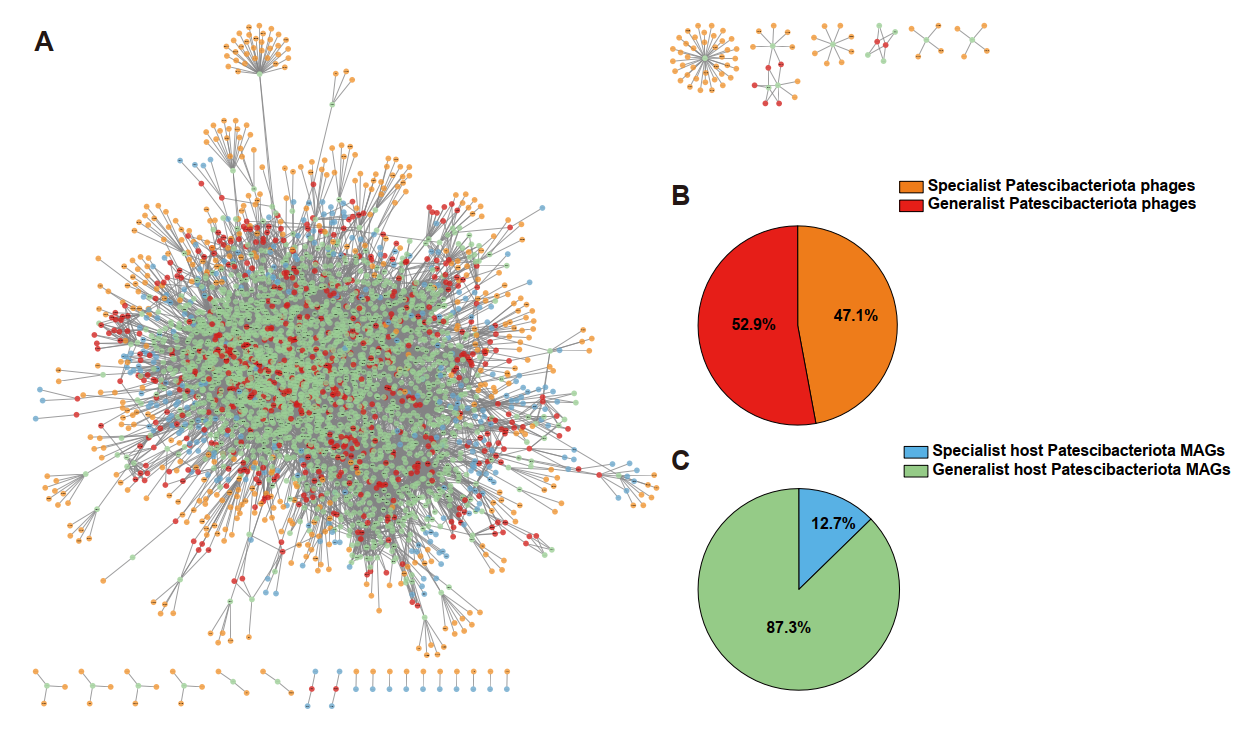


Figure S4. Interactions between host Patescibacteriota MAGs and their phages in groundwater ecosystems. (A) Schematic illustration depicting the 14120 pairs phage-Patescibacteriota interactions. The orange and red nodes represent specialist and generalist Patescibacteriota phages, respectively. The blue and green nodes represent specialist and generalist host Patescibacteriota MAGs, respectively. (B) Relative proportion of specialist and generalist Patescibacteriota phages. (C) Relative proportion of specialist and generalist host Patescibacteriota MAGs.


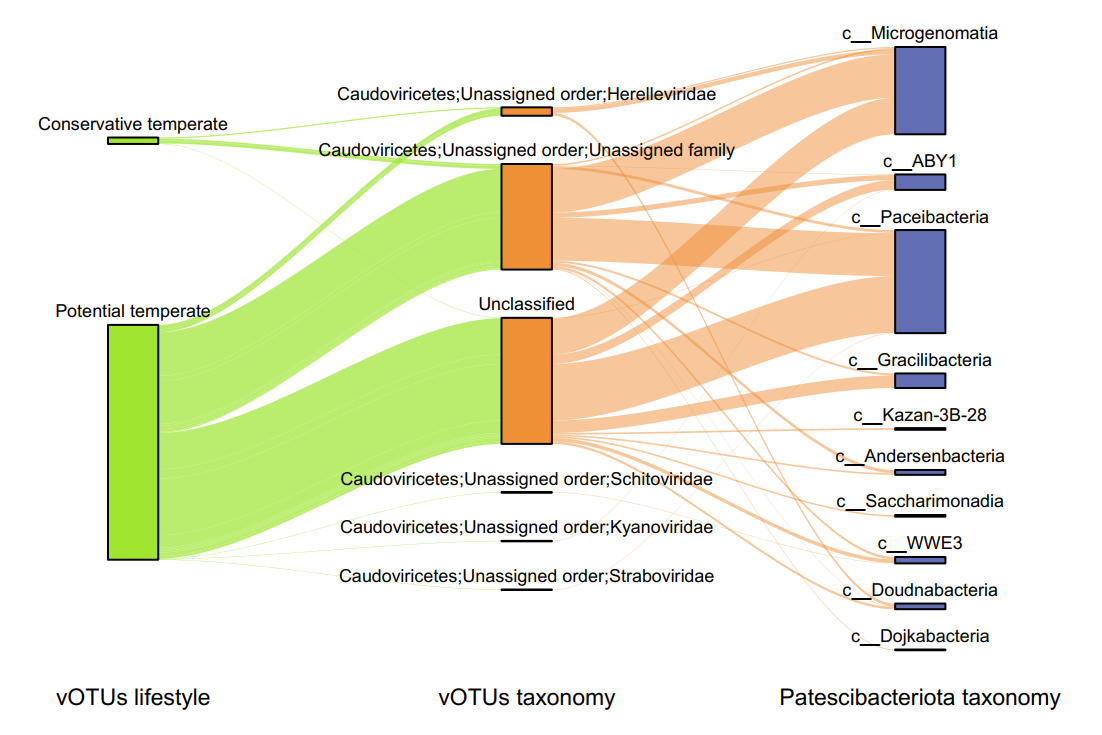


Figure S5. Assignment of temperate vOTUs to host Patescibacteriota MAGs based on nucleotide sequence homology search.


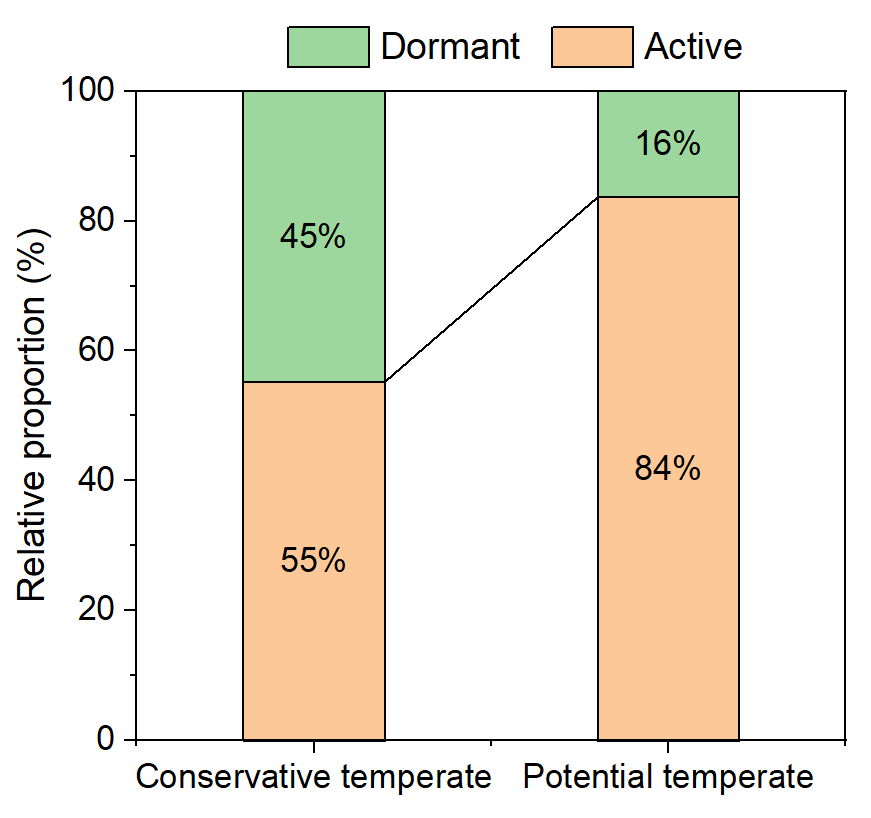


Figure S6. Potential activity of temperate Patescibacteriota vOTUs.


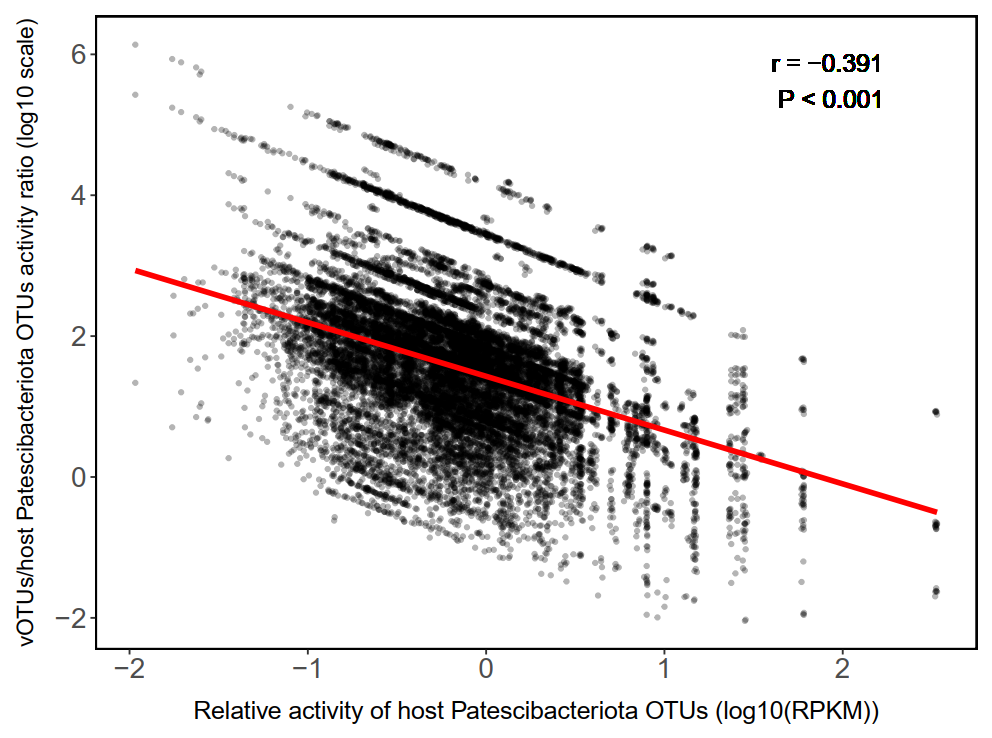


Figure S7. Correlation between the relative activity of host Patescibacteriota OTUs and vOTUs/host Patescibacteriota OTUs activity ratios.


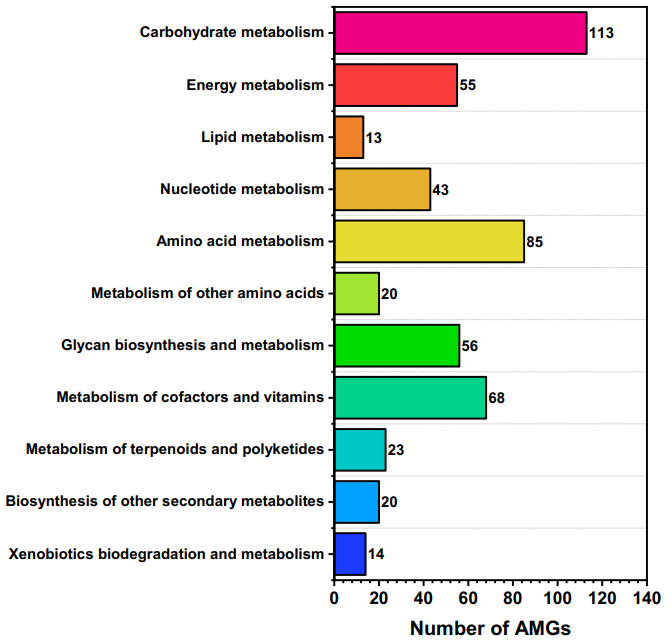


Figure S8. Functional annotations of auxiliary metabolic genes (AMGs) encoded by groundwater Patescibacteriota phages based on KEGG database.


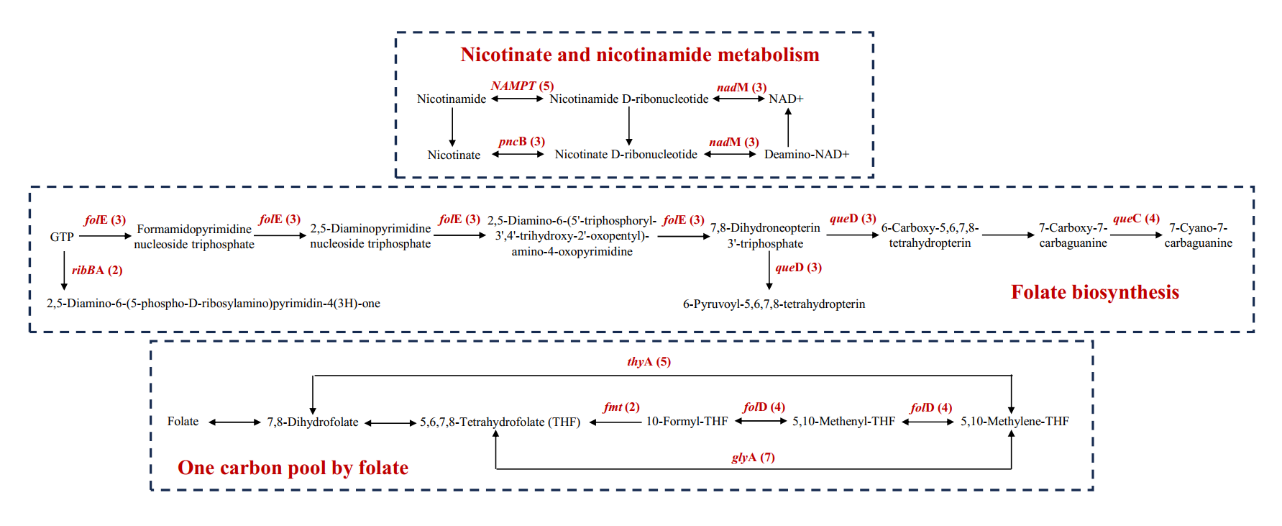


Figure S9. Putative auxiliary metabolic genes (AMGs) identified in groundwater Patescibacteriota phages involved in nicotinate and nicotinamide metabolism, folate biosynthesis, and one carbon pool by folate. The phage-encoded AMGs are highlighted in red within these pathways, with Arabic numerals in parentheses indicating their respective quantities.


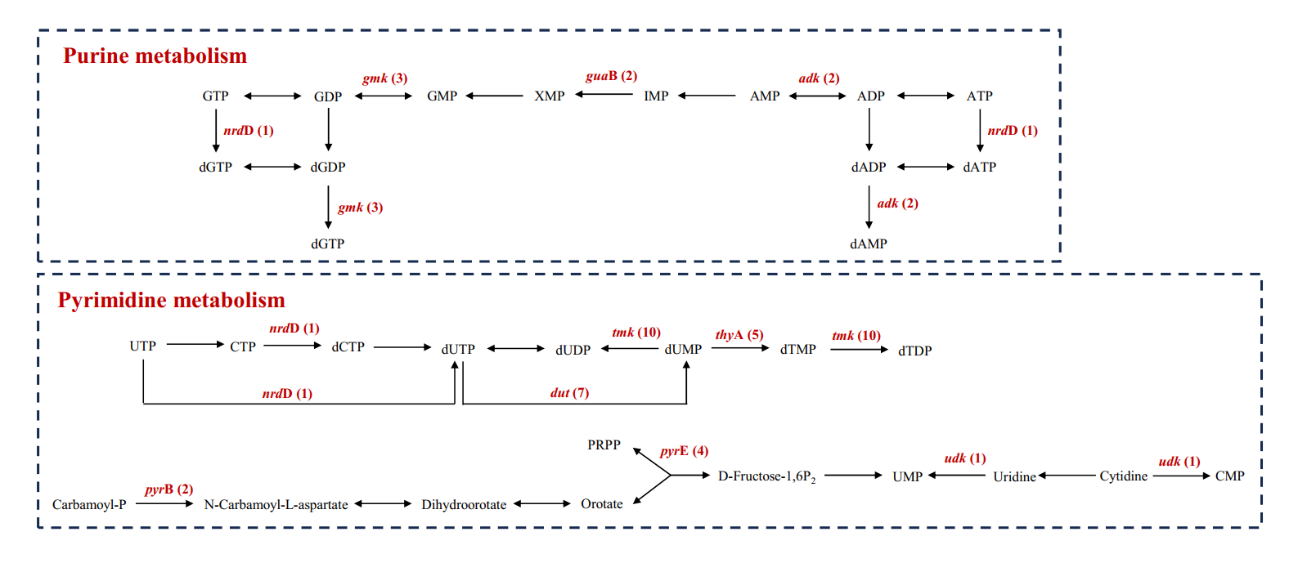


Figure S10. Putative auxiliary metabolic genes (AMGs) identified in groundwater Patescibacteriota phages involved in nucleotide metabolism. The phage-encoded AMGs are highlighted in red within these pathways, with Arabic numerals in parentheses indicating their respective quantities.


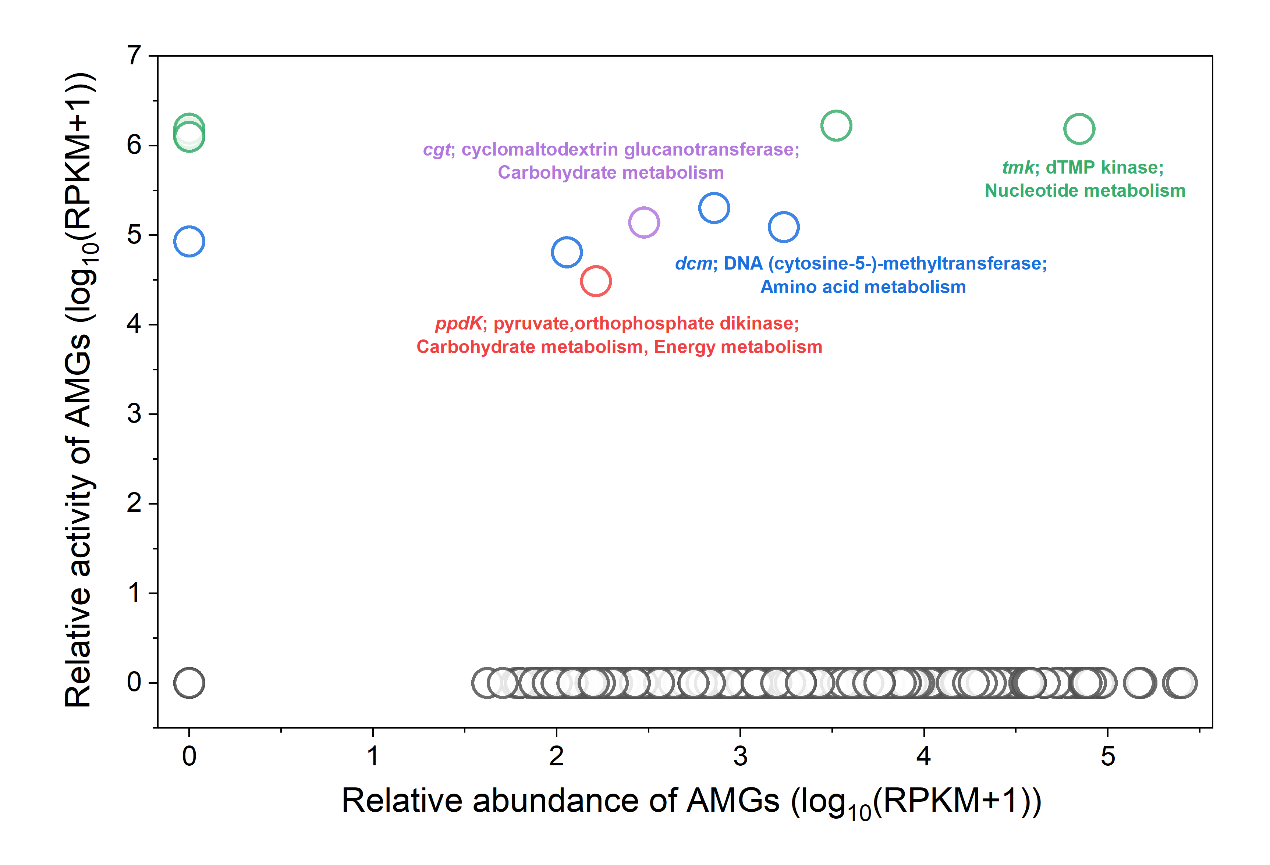


Figure S11. The relative activity and the relative abundance of all auxiliary metabolic genes (AMGs) identified in this study (carried by the groundwater Patescibacteriota phages) across six metatranscriptomic datasets and their corresponding metagenomic datasets from the Colorado site. Functional annotations of selected AMGs are indicated with same-color text labels.

# Supplementary Tables

Table S1. Putative auxiliary metabolic genes (AMGs) identified in groundwater Patescibacteriota phages associated with transport system.

|  | ID | Description | Count |
| --- | --- | --- | --- |
| P-type transporter | K01531 | P-type Mg^2+^ transporter [EC:7.2.2.14] | 1 |
|  | K01533 | P-type Cu^2+^ transporter [EC:7.2.2.9] | 4 |
|  | K01537 | P-type Ca^2+^ transporter type 2C [EC:7.2.2.10] | 5 |
|  | K17686 | P-type Cu^+^ transporter [EC:7.2.2.8] | 2 |
| ABC transport system | K01990 | ABC-2.A; ABC-2 type transport system ATP-binding protein | 1 |
|  | K02003 | putative ABC transport system ATP-binding protein | 1 |
|  | K02004 | putative ABC transport system permease protein | 2 |
|  | K01999 | *liv*K; branched-chain amino acid transport system substrate-binding protein | 1 |
|  | K02027 | ABC.MS.S; multiple sugar transport system substrate-binding protein | 1 |
|  | K02032 | peptide/nickel transport system ATP-binding protein | 1 |
|  | K02033 | ABC.PE.P; peptide/nickel transport system permease protein | 1 |
|  | K02035 | ABC.PE.S; peptide/nickel transport system substrate-binding protein | 1 |
|  | K10823 | oligopeptide transport system ATP-binding protein | 1 |
|  | K09811 | cell division transport system permease protein | 10 |
|  | K09812 | cell division transport system ATP-binding protein | 9 |
